# Green algae in alpine biological soil crust communities: acclimation strategies against ultraviolet radiation and dehydration

**DOI:** 10.1007/s10531-014-0653-2

**Published:** 2014-03-02

**Authors:** Ulf Karsten, Andreas Holzinger

**Affiliations:** 1 Institute of Biological Sciences, Applied Ecology and Phycology, University of Rostock, Albert-Einstein-Strasse 3, 18059 Rostock, Germany; 2 Functional Plant Biology, Institute of Botany, University of Innsbruck, Sternwartestraße 15, 6020 Innsbruck, Austria

**Keywords:** Antioxidants, Biodiversity, *Klebsormidium*, Organic osmolytes, Ultrastructure, UV-sunscreens

## Abstract

Green algae are major components of biological soil crusts in alpine habitats. Together with cyanobacteria, fungi and lichens, green algae form a pioneer community important for the organisms that will succeed them. In their high altitudinal habitat these algae are exposed to harsh and strongly fluctuating environmental conditions, mainly intense irradiation, including ultraviolet radiation, and lack of water leading to desiccation. Therefore, green algae surviving in these environments must have evolved with either avoidance or protective strategies, as well as repair mechanisms for damage. In this review we have highlighted these mechanisms, which include photoprotection, photochemical quenching, and high osmotic values to avoid water loss, and in some groups flexibility of secondary cell walls to maintain turgor pressure even in water-limited situations. These highly specialized green algae will serve as good model organisms to study desiccation tolerance or photoprotective mechanisms, due to their natural capacity to withstand unfavorable conditions. We point out the urgent need for modern phylogenetic approaches in characterizing these organisms, and molecular methods for analyzing the metabolic changes involved in their adaptive strategies.

## Introduction

Biological soil crusts (BSCs) are formed by various groups of living organisms and their by-products, creating a millimeter-thick topsoil layer of inorganic particles bound together by organic materials. BSCs occur on all continents on Earth, in warm arid and semi-arid habitats as well as in other climate zones such as alpine regions, where soil moisture is limiting and cover provided by higher plants is sparse (Belnap and Lange 2001). Along with other microorganisms such as heterotrophic bacteria, archaea and fungi, as well as with macroscopic lichens and bryophytes, cyanobacteria and algae are the most important phototrophic components of BSCs (Elbert et al. 2012). These communities can be characterized as “ecosystem engineers” forming water-stable aggregates that have important, multifunctional ecological roles in primary production, nitrogen (N) cycling, mineralization, water retention, and stabilization of soils (Evans and Johansen 1999; Lewis 2007; Reynolds et al. 2001). A recent review on BSCs clearly demonstrated their important ecological contribution to global carbon (C) fixation (about 7 % of terrestrial vegetation) and nitrogen (N) fixation (about 46 % of terrestrial biological N fixation) (Elbert et al. 2012). Although the ecological structure and function of BSC communities from subtropical to polar regions have been studied in recent decades (Belnap and Lange 2001; Büdel 2005), less is known about similar communities living in high alpine habitats such as the Alps (Türk and Gärtner 2001).

BSCs from the Alps have been described from bare mineral soils, soil gaps between higher plants, underneath higher plants, peat, plant debris, and even on fluvioglacial deposits up to the nival zone (Ettl and Gärtner 1995; Reisigl 1964; Türk and Gärtner 2001). However, most studies on aeroterrestrial algae have focused on classical systematics (Ettl and Gärtner 1995). Soil algae of alpine habitats are members of various groups of the Xanthophyta, Eustigmatophyta, Chlorophyta and Streptophyta; in this review we focus on green algae from the last two divisions.

## Environmental conditions for alpine biological soil crust communities

In the Alps, a relatively large proportion of the landscape lies in the subalpine, alpine and nival zones. Here the abiotic conditions show dramatic gradients and extensive patterns of small-scale habitats (Körner 2003; Larcher 2012). Over short elevational distances, the thermal gradients reflect the climate across vast latitudinal distances, resulting in a compression of life zones (Körner 2003; Larcher and Wagner 2009). The steep abiotic gradients include wide diurnal temperature fluctuations, occasional frost in summer, intense irradiation even at low temperatures, a large increase in ultraviolet radiation (UVR) with altitude, and high impacts by wind or storms that produce short-term drought and abrasion. Therefore, high mountains are extreme habitats, which set selective boundaries/limits to the altitudinal distributions of BSCs. In addition to the altitudinal gradients, the chemistry of the underlying rocks (e.g., limestone or silicate) influences soil formation and properties (e.g., pH value), and consequently the settlement and ecology of all primary producers. Organisms living in alpine regions must be well adapted to these extreme conditions to assure their long-term survival. Among the most extreme microorganisms in alpine regions are snow and ice algae (for summary see Remias 2012).

Two major abiotic factors affect alpine BSC algae in particular. The first is the periods of dehydration, which slow metabolic processes. Dehydration is followed by desiccation, leading to a total cessation of metabolic processes. The second prominent abiotic factor is exposure to UVR. In the Alps, water availability frequently fluctuates, from fluid droplets after rain or snow, to extended periods of dryness or freezing. Water availability, which includes precipitation, condensation and water vapor, is therefore the key ecological prerequisite for long-term survival of aeroterrestrial algae, because only fully hydrated and ultrastructurally intact cells are physiologically functional (for summary see Holzinger and Karsten 2013). Comparisons with, e.g., Antarctic wetlands could be drawn, where low subzero temperatures lead to annual winter freezing. These extreme cold periods caused little harm to cyanobacteria, but were fatal to 50 % of the algal population (Šabacká and Elster 2006).

The Alps are among the regions with the highest UVR levels recorded for Europe. Solar radiation entering the Earth’s atmosphere exhibits a typical spectrum characterized by UVR (190–400 nm), photosynthetically active radiation (PAR: 400–700 nm) and infrared radiation (IR: >700). UVR is differentiated according to the CIE definition into three wavebands—UV-C: 190–280 nm, UV-B: 280–315 nm, and UV-A: 315–400 nm. Due to the absorption features of stratospheric ozone, the intensity of radiation in the UV-B range is globally increasing, because of the destruction of the stratospheric ozone. Besides clouds, atmospheric particles and snow-covered surfaces, changes in day length, season, latitude and altitude produce wide variability in the radiation conditions of terrestrial ecosystems. Particularly, the altitude effect is very well documented for the European Alps (Blumthaler et al. 1996; Blumthaler 2012). These authors showed that under a clear sky in summer, UV-A increases by about 9 % per 1,000 m and UV-B by 18 % per 1,000 m. In addition, Blumthaler and Ambach (1990) found evidence for an increasing trend of UV-B in the Alps, due to stratospheric ozone depletion. Consequently, high-alpine ecosystems and their communities such as BSCs experience seasonally fluctuating enhanced desiccation and UVR conditions. While adaptive strategies in higher plants of the Alps and other mountains have been intensively studied (Larcher 2003; Körner 2003; Holzinger et al. 2007; Lütz and Engel 2007, and references therein), corresponding data on BSC algae from these areas are still very limited (Türk and Gärtner 2001; Karsten et al. 2010, 2013; Karsten and Holzinger 2012), but particularly interesting, as UVR can act as a destructive factor on exposed green algae (Holzinger and Lütz 2006).

## Diversity of algae in alpine biological soil crust communities

Eukaryotic soil algae of mountains are represented mainly by monadoid and coccoid green algae (Chlorophyta), various groups of the Xanthophyceae, as well as by the filamentous genus *Klebsormidium* (Streptophyta) (Gärtner 2004; Ettl and Gärtner 1995; Tschaikner et al. 2007, and references therein). Reisigl (1964) was the first to undertake a systematic survey on aeroterrestrial algae in alpine soils of the Tyrolean Alps above 3,000 m a.s.l. Using a morphological approach, Reisigl described 89 species with 28 taxa belonging to the Xanthophyceae. A decade later, Vinatzer (1975) investigated soil algae in the South Tyrolean Dolomites (Italy) and reported 77 species (16 Xanthophyceae). Although other algal taxa such as members of the Bacillariophyceae, Chrysophyceae, Dinophyceae etc. are regularly described from alpine soils (Ettl and Gärtner 1995), the most abundant and dominant organisms are green algae (Chlorophyta, Streptophyta). This pattern was repeated in various investigations of BSC algae from North American deserts (Cardon et al. 2008; Lewis and Lewis 2005; Lewis 2007), which indicated that mainly green algae are present in these soil communities. These authors documented that although green microalgae from soils appear morphologically simple and similar, they are genetically extraordinarily diverse, with their membership spanning at least five green-algal classes and encompassing many new, still undescribed taxa. To date, at least several hundred taxa of unicellular green algae have been cultured and phylogenetically analyzed using 18S rDNA sequence data from desert BSC samples. However, a molecular-taxonomic approach with modern sequencing techniques for the evaluation of the biodiversity of alpine BSC algae is completely missing. Only individual alpine isolates have been characterized by large subunit *rbc*L or ITS-1 and ITS-2 rDNA sequencing (Kaplan et al. 2012; Karsten et al. 2013). Therefore, we expect a much higher species number, as previously noted in conjunction with cryptic biodiversity (Reisigl 1964; Vinatzer 1975). Moreover, an ecological differentiation among cryptic species of *Klebsormidium* was suggested recently by Škaloud and Rindi (2013), and these species might also have preferences for certain substrata.

## Ultraviolet radiation stress in biological soil crust algae

Solar radiation is essential for all phototrophic life on Earth. An increase in UVR, however, can inhibit many biological processes. The major cellular targets of UV-B are various biomolecules that directly absorb this waveband, such as DNA and proteins, or that are indirectly affected by various UV-induced photochemical reactions. The biological and, finally, the ecological consequences are manifold.

DNA is one of the most UV-sensitive biomolecules; UV-induced damage occurs directly by the absorption of UV-B quanta through the aromatic residues. The structural consequences are conformational alterations such as the often-observed formation of cyclobutane dimers and pyrimidine (6-4)-pyrimidone (6-4)-photoproducts (Lois and Buchanan 1994). This UV-induced DNA damage can significantly compromise the accuracy of nucleic acid transcription and replication, causing misreading or erroneous replication, which is reflected in an increasing number of mutations. A higher mutation rate will eventually result in reduced gene expression and hence debilitation or even increased mortality of algal cells.

UV-B induced damage to proteins is mediated by aromatic amino acids or by disulfide bonds between cysteine residues, which can be easily cleaved after absorption of this waveband (Vass 1997). Typical target proteins in algae are those involved in photosynthesis, such as the D1 protein of photosystem II (PSII) and the enzyme Rubisco in the Calvin cycle (Campbell et al. 1998; Bischof et al. 2000); damage to these results in decreased photosynthetic activity and growth. However, since proteins typically occur as numerous copies inside the algal cell, any UV-induced damage to proteins is not as severe as the damage to DNA (Harm 1980).

UV-B-induced photo-oxidative stress stimulates various cellular processes, leading to the production of reactive oxygen species (ROS) such as superoxide radicals and hydrogen peroxide, as well as singlet-oxygen and hydroxyl radicals. The sources and production sites of ROS are mainly related to photosynthetic activities such as pseudocyclic photophosphorylation and the Mehler reaction, which stimulate the accumulation of hydrogen peroxide (Asada 1994; Elstner 1990). UV-induced ROS are extremely toxic to algal cells, by causing oxidative damage to all biomolecules, particularly lipids. After a first initiation reaction, an unsaturated fatty acid is converted to a peroxyl radical, which in turn attacks another unsaturated fatty acid, finally leading to some kinds of free-radical cascades. This photochemical peroxidation of unsaturated fatty acids may be particularly damaging to membrane structure and function (Bischof et al. 2006).

As a consequence of UV-induced damage to biomolecules, many physiological processes are potentially impaired. Photosynthesis is probably the most intensively studied process in plant sciences. Due to its biochemical complexity, numerous sites can be affected by UV-B. These can include inhibition of energy transfer within the PSII reaction center, the water-splitting complex, or the light-harvesting complex. Key enzymes such as Rubisco and ATPase are also typical targets. The common consequences of UV-B for photosynthetic function are decreased or even fully inhibited CO_2_-fixation, and hence a decline in primary production (Franklin and Forster 1997; Bischof et al. 2006). Nevertheless, the extent to which alpine BSC algae are affected by UVR is not well understood.

**Fig. 1:**
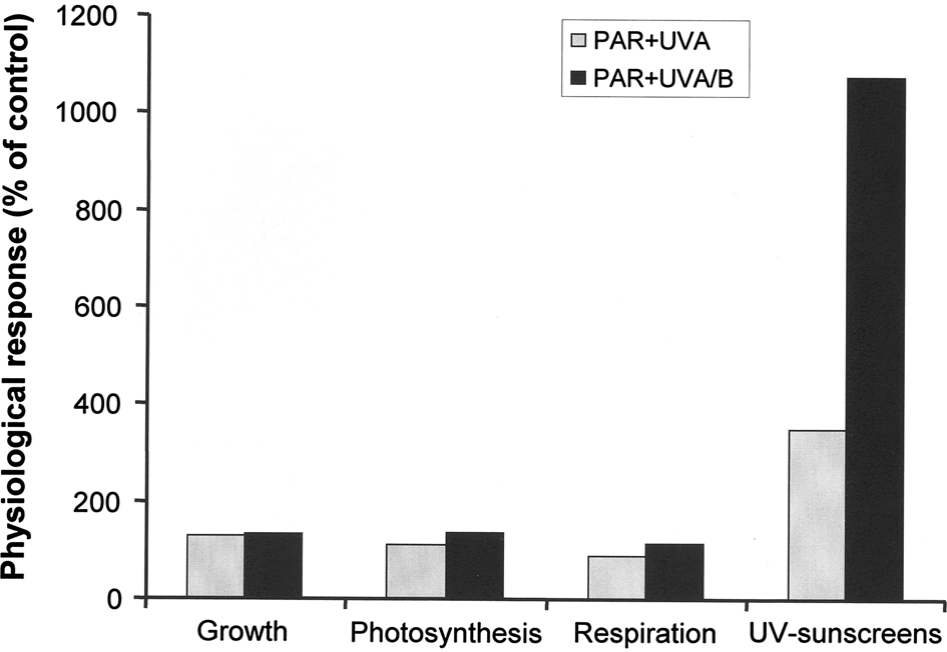
The effect of PAR+UV-A and PAR+UV-A/B on growth, photosynthesis, respiration, and the capability to synthesize and accumulate UV-sunscreen compounds in the alpine biological soil crust green alga *Klebsormidium dissectum* strain ASIB V103. This species was isolated at 2,363 m a.s.l. (Pitschberg, St. Ulrich in Gröden, South Tyrol, Italy). The physiological responses are expressed as relative percentages in relation to the control (PAR, 100 %)

The filamentous green alga *Klebsormidium fluitans*, strain ASIB V103, was isolated from a BSC underneath a stand of the grass *Festuca rubra* at 2,363 m a.s.l. (Pitschberg, St. Ulrich in Gröden, South Tyrol, Italy). In the laboratory, *K. fluitans* was exposed under controlled conditions for up to 10 days to different defined radiation scenarios P, PA and PAB (P: 47 μmol photons m^−2^ s^−1^ PAR; PA: 50 μmol photons m^−2^ s^−1^ PAR + 5.0–6.3 W m^−2^ UV-A; PAB: 55 μmol photons m^−2^ s^−1^ PAR + 7.3–9.2 W m^−2^ UV-A + 0.4–0.5 W m^−2^ UV-B), according to the methodology described by Karsten et al. (2007). The data clearly indicated that growth, photosynthesis and respiration were not affected by both UV-A and UV-B, and were even slightly stimulated (Fig. 1), indicating a high UVR tolerance.

**Fig. 2:**
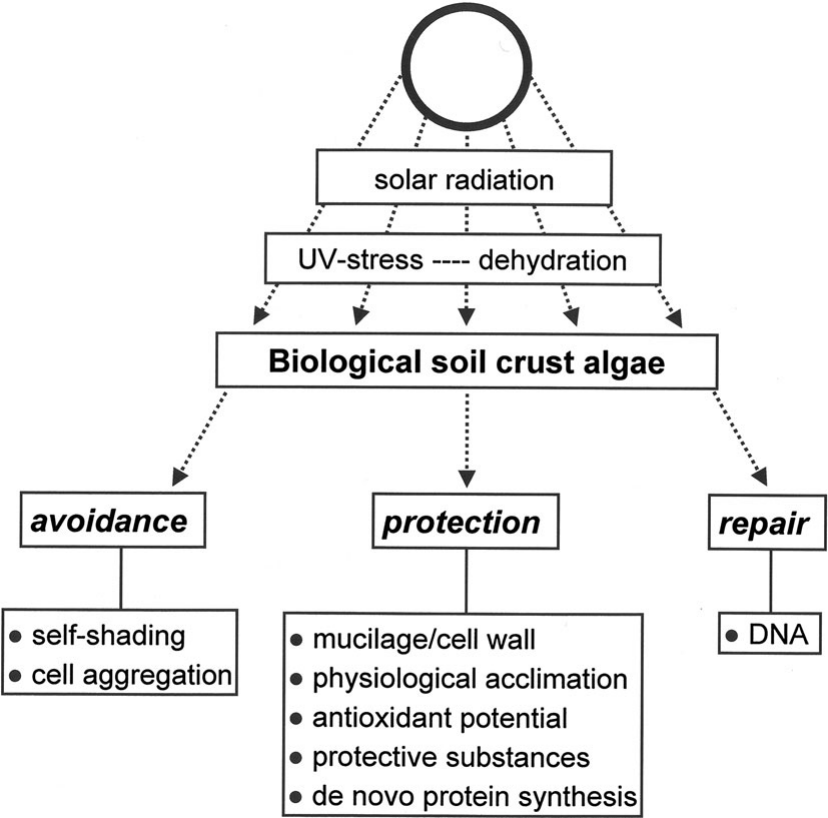
Strategies of alpine biological soil crust algae to counteract biologically harmful UV radiation and dehydration

If BSC algae are confronted with UVR in their natural habitats, they rely on several different strategies to mitigate or even prevent biologically harmful UV-effects and assure long-term survival. These include avoidance, numerous protective mechanisms, and repair of DNA, which is demonstrated in a summary scheme (Fig. 2). BSC algae typically occur in a matrix of polymeric organic and inorganic substances, and in association with other organism groups. In BSC of North American deserts, green algae occupy microenvironments within the crust matrix, where they are protected from damaging radiation levels and exposure to drying atmosphere (Gray et al. 2007). These data clearly show that self-shading by surrounding cells or filamentous algae inside BSCs is an important protective mechanism. Under natural conditions the filamentous BSC green alga *Klebsormidium* often forms multi-layered mat-like structures on top of or interwoven with the upper millimeters of soil, which contribute to a high degree of self-shading as a passive photoprotective mechanism (“umbrella”) for individual filaments inside such a population (Karsten et al. 2010). Similarly, in the semi-terrestrial green algal genus *Zygnema*, thick mat-like layers survive experimentally generated high UVR to PAR ratios by self-shading (Holzinger et al. 2009; Pichrtová et al. 2013). In addition, the formation of spores and other permanent stages (such as akinetes) may contribute to coping with enhanced UVR (for summary see Holzinger and Lütz 2006).

The response of any alga to UV-B exposure is determined by the interplay of genetically fixed adaptation and physiological acclimation (Bischof et al. 2006). While the UVR-tolerance mechanisms of marine algae are very well studied, adequate data on alpine BSC algae are still missing. Many algae can cope with intense radiation in summer, due to their ability for dynamic photoinhibition, a photoprotective mechanism by which excessive energy absorbed is rendered harmless by thermal dissipation (Krause and Weiss 1991). Effective ROS elimination by antioxidants (vitamins c, e, glutathione) and/or antioxidative enzymes (catalase, superoxide-dismutase, etc.), DNA repair by photolyase and de-novo biosynthesis of damaged proteins are well-described protective mechanisms (Bischof et al. 2006). For alpine BSC algae exposed to UVR for substantial parts of their life cycles, strategies that passively screen this harmful waveband will contribute to preventing UV-induced direct and indirect damage to essential biomolecules. In addition, UV screening may also save metabolic energy by reducing the need for constantly active avoidance and repair processes.

The most common photoprotective sunscreens in many, but not all algal taxa studied thus far are the mycosporine-like amino acids (MAAs), a suite of chemically closely related, colorless, water-soluble, polar and (at cellular pH) uncharged or zwitterionic amino acid derivatives. Most of the ~25–30 described MAAs are derivatives of an aminocyclohexenimine structure that absorbs maximally at UV-A/B wavelengths. These molecules were presumed to function as passive shielding solutes by dissipating the absorbed UVR energy in the form of harmless heat without generating photochemical reactions. MAAs exhibit extremely high molar absorptivity for UV-A and UV-B (molar extinction coefficients between 28,000 and 50,000), and have been reported as photochemically stable structures, both of which are prerequisites for their sunscreen function (Bandaranayake 1998).

In the alpine BSC alga *K. fluitans* strain ASIB V103, the presence of a unique MAA and its response patterns under UVR have been investigated. This isolate contained one specific, but chemically not elucidated MAA with an absorption maximum at 324 nm. Exposure to UV-A and UV-B led to an almost 4- and 11-fold, respectively, increase in the MAA concentration (Fig. 1). Under UV-B this MAA contributed almost 1 % of the dry weight, a somewhat higher proportion compared to other sunscreens or pigments. The biochemical capability to synthesize and accumulate high MAA concentrations under UVR stress explains the rather UV-insensitive growth, photosynthesis and respiration in *K. fluitans* (Fig. 1). In contrast, another alpine semi-terrestrial green alga from the family Zygnematophyceae, *Zygogonium ericetorum*, lacks MAA but contains other compounds involved in UVR protection such as specific phenolics and hydrolyzable tannins (Aigner et al. 2013).

## Dehydration stress in biological soil crust algae

The loss of water from an algal cell causes severe, often lethal stress (e.g. Büdel 2011), because the chemical structure of all biomolecules and membranes is maintained by water molecules. Dehydration leads to the often irreversible aggregation of macromolecules and the subsequent disintegration of organelles, resulting in loss of their functions. Similarly to UV-B stress, the biological and ecological consequences are manifold.

Water loss suppresses photosynthesis in alpine and desert BSC green algae (Gray et al. 2007; Karsten et al. 2010; Karsten and Holzinger 2012). For example, unialgal cultures of BSC green algae from deserts can survive at least 4 weeks under controlled conditions (Gray et al. 2007). The survival and activity rates were investigated in members of several genera including *Bracteacoccus* sp., *Scenedesmus rotundus*, *Chlorosarcinops*is sp., *Chlorella* sp. and *Myrmecia* sp. by Gray et al. (2007). They showed that dehydration-tolerant desert algae and closely related aquatic relatives differed widely in the recovery kinetics of photosynthesis after rewetting; the desert lineages recovered much faster than their aquatic relatives. Furthermore desert algae survived desiccation for at least 4 weeks when dried out in darkness, and recovered to high levels of photosynthetic quantum yield within 1 h of rehydration in darkness (Gray et al. 2007). The process of desiccation has also been studied extensively in the chlorophyte partners of lichens, e.g., *Trebouxia*; these algae react differently in resurrection, depending on whether they were dehydrated slowly or rapidly prior to the desiccation phase (Gasulla et al. 2009). In addition, temperature might play a crucial role, as recently demonstrated in the changeover between two *Microcoleus* species across different temperature gradients in the southern deserts of the USA (Garcia-Pichel et al. 2013).

**Fig. 3:**
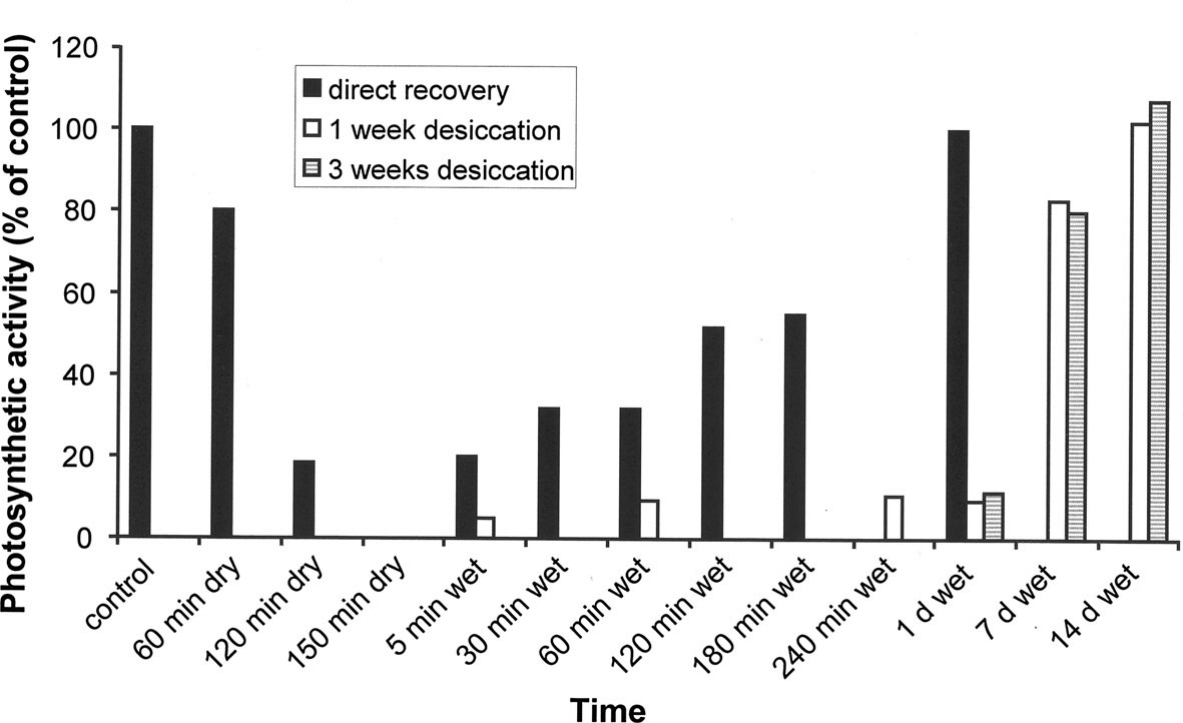
Changes in photosynthetic activity (Fv/Fm, optimum quantum yield) in the alpine biological soil crust green alga *Klebsormidium dissectum* (SAG 2416) during short-term (<2.5 h) and long-term desiccation (1, 3 weeks), as well as during the recovery phase after rehydration. This species was isolated at 2,350 m a.s.l. (Schönwieskopf, Obergurgl, Tyrol, Austria). The photosynthetic responses are expressed as relative percentages in relation to the control (100 %). Figure modified after Karsten et al. (2013)

**Fig. 4:**
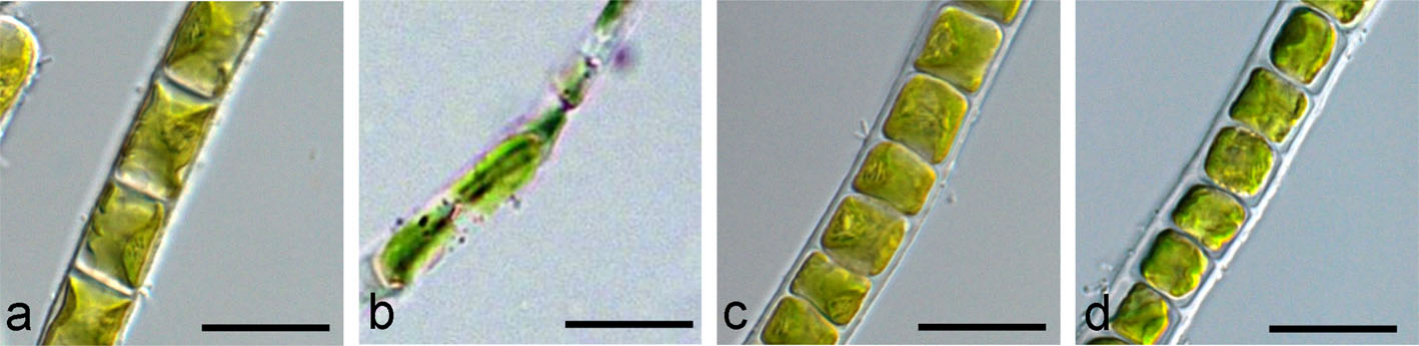
Light micrographs of *Klebsormidium crenulatum* (SAG 2415), **a** control cells, **b** desiccated at 5 % air relative humidity for 1 day, **c** plasmolysed in 800 mM sorbitol, **d** plasmolysed in 2,000 mM sorbitol. **b** desiccated sample viewed in immersion oil, contraction of the whole filament visible, **c** incipient plasmolysis, **d** advanced plasmolysis. *Bars* 10 μm. **a**, **c**, **d** reprinted from Kaplan et al. (2012) with permission of Springer Science and Business Media; **b** reprinted from Holzinger et al. (2011) with permission of the Phycological Society of America

A similar high tolerance of dehydration is present in some alpine BSC algae (Fig. 3). The green alga *Klebsormidium dissectum* was isolated from the top 5 mm of an alpine BSC collected at 2,350 m a.s.l. (Schönwieskopf, Obergurgl, Tyrol, Austria, Karsten and Holzinger 2012) and deposited in the Göttingen culture collection (SAG 2416). This species was air-dried for 2.5 h under controlled conditions, and photosynthesis (measured as optimum quantum yield) continuously decreased, eventually reaching a state of complete inhibition within this time period (Fig. 3). Subsequent rehydration was accompanied by moderate recovery kinetics, i.e., although after 3 h about 55 % of the control activity could be measured, almost 1 day was necessary for complete restoration of photosynthetic activity. In contrast, desiccation for 1 and 3 weeks, respectively, led to a lengthy delay in the recovery kinetics. Periods of 7–14 days were necessary for photosynthesis to reach the original level of the control (Fig. 3). This is likely due to a higher rate of lethality under prolonged desiccation, which was estimated to be ~80 % after 2 day at 5 % relative humidity (RH) (Karsten and Holzinger 2012). Similar results were described for *Klebsormidium crenulatum* (Fig. 4a; Holzinger et al. 2011), which coexisted with *K. dissectum* in the alpine BSCs at Obergurgl, Austria (Karsten et al. 2010; Göttingen, SAG 2415). However, there were differences in the desiccation tolerance of the two species of *Klebsormidium*, which can be explained by differences in their morphological and structural features (Karsten et al. 2010; Holzinger et al. 2011; Karsten and Holzinger 2012). While *K. crenulatum* forms rather long, strong filaments, sometimes growing in rope-like aggregates that support high self-protection against water loss, the coexisting *K. dissectum* has smaller filaments that easily disintegrate.

Since in the dehydrated state, photosynthesis would be completely blocked, any further excitation energy absorbed cannot be used for electron transport, and hence may result in photoinhibition or even photodamage (Wieners et al. 2012). Various desiccation-sensitive sites in the photosynthetic apparatus have been reported: the photosystems, particularly PSII with its oxygen-evolving complex, ATP generating, and carbon assimilation processes (Allakhverdiev et al. 2008; Holzinger and Karsten 2013).

Although dehydration effects on the CO_2_ exchange in alpine BSC algae have to our knowledge not been reported in the literature, there exist some data on the aeroterrestrial green alga *Apatococcus lobatus*, one of the most abundant taxa in temperate Europe, which forms conspicuous biofilms on trees and building surfaces (Gustavs et al. 2011). This species forms cell packets surrounded by mucilage, thereby achieving hydration equilibrium with the vapor pressure of the atmosphere (Bertsch 1966). The maximum carbon assimilation in *A. lobatus* was determined at 97–98 % RH, while at 90 % RH, 50 % of the maximum CO_2_-uptake was measured. The lower limit of carbon assimilation was estimated at 68 % RH (Bertsch 1966). These data clearly indicated that atmospheric moisture favors CO_2_-uptake in *A. lobatus*, compared to liquid water, which inhibits uptake. The water content of *Klebsormidium flaccidum* also determines the carbon dioxide supply and hence the photosynthetic rate (De Winder et al. 1990). While reduced water content due to dehydration leads to inhibition of photosynthesis if species-specific thresholds fall below, high water content (i.e., supersaturation, for example, after rainfall) typically limits CO_2_ diffusion into the cells, also resulting in the inhibition of photosynthesis. The CO_2_-exchange mechanism in *Apatococcus*, and most probably also in alpine BSC algae, likely mirrors the adaptations of alpine BSC algae that exist in a terrestrial environment.

Ecophysiological studies of many plants indicate that photosynthesis and respiration exhibit different responses when dehydrated, and that photosynthesis is less tolerant than respiration to many environmental stresses. An explanation of the different susceptibility of the two physiological processes may be related to the structural properties of chloroplasts and mitochondria. While chloroplasts easily swell or shrink depending on intracellular water content, with consequences for the thylakoid fine structure, functionally the location of the photosynthetic electron transport chain affects the mitochondrial cristae ultrastructure less (Kirst 1990).

Physiological constraints caused by dehydration in BSC green algae were mainly investigated in relation to photosynthesis (see above), and hence far less is known about molecular and cell biological changes that accompany water loss.

## Structural and ultrastructural features of alpine biological soil crust algae

**Fig. 5:**
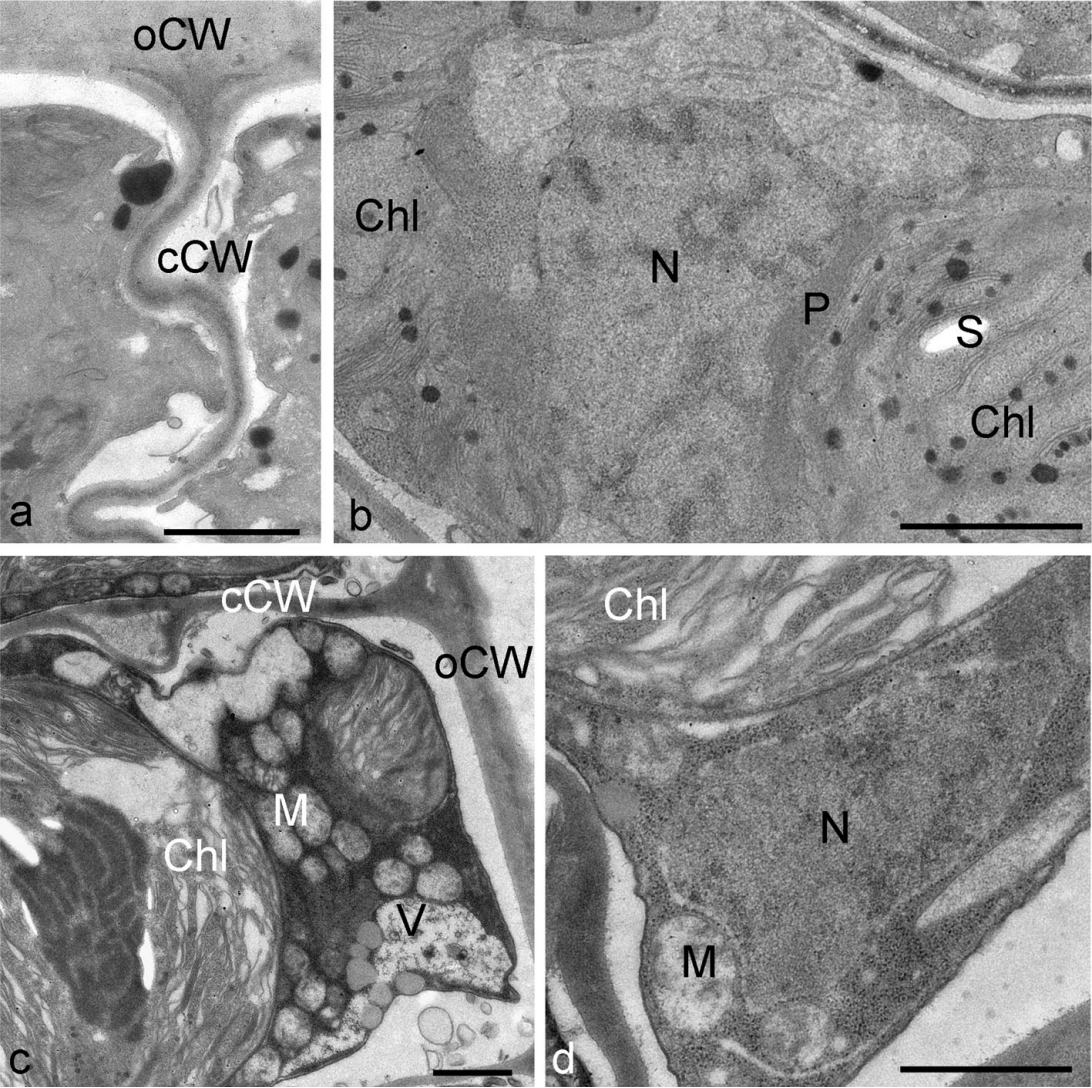
Transmission electron micrographs of *Klebsormidium crenulatum* (SAG 2415), **a** desiccated at 95 % air relative humidity for 4 days, **b** desiccated at 5 % air relative humidity for 4 days, **c**, **d** plasmolysed with 1,000 mM sorbitol for 3 h. The general appearance of the cytoplasm is similarly dense regardless of the different treatments, except that in desiccated samples the cross walls appear undulated (**a**). *oCW* outer cell wall, *cCW* cross cell wall, *Chl* chloroplast, *M* mitochondrion, *N* nucleus, *P* peroxisome, *S* starch, *V* vacuole. *Bars* 1 μm. **a**, **b** reprinted from Holzinger et al. (2011) with permission of the Phycological Society of America; **c**, **d** reprinted from Kaplan et al. (2012) with permission of Springer Science and Business Media

Limited data on the structure and ultrastructure of alpine BSC algae are available. This scarcity of information is most likely due to the limited availability of taxonomically characterized algae from these habitats (e.g., Tschaikner et al. 2007, 2008; Holzinger et al. 2011; Karsten and Holzinger 2012). Characterization of whole soil crusts has been attempted by scanning electron microscopy (e.g., Hoppert et al. 2004; Büdel 2005). Microscopic observation of desiccated cells has been recently achieved for *K. crenulatum* (Fig. 4b; Holzinger et al. 2011). Additionally, water loss has been generated by exposure to hyperosmotic solutions in *Klebsormidium* (Fig. 4c, d; Kaplan et al. 2012). Ultrastructural changes as a consequence of desiccation have been reported earlier in field-collected *Klebsormidium* (Morison and Sheath 1985) and another crust-forming green alga, *Zygogonium* (Hoppert et al. 2004; Holzinger et al. 2010), as well as in alpine BSC algae and alpine algae from semi-terrestrial habitats (Holzinger et al. 2011; Karsten et al. 2010; Karsten and Holzinger 2012; Aigner et al. 2013; Kaplan et al. 2012, 2013). In these algae the basic organelles such as the nucleus, chloroplast and mitochondria remain intact upon desiccation, and the cytoplasm appears extremely condensed (Fig. 5a, b). Elementary differences were found in the cell walls of these genera. While in *Klebsormidium* the secondary walls remain flexible and have a good capacity to follow the shrinkage process (Holzinger et al. 2011; Karsten and Holzinger 2012), the cell walls of *Zygogonium* are thick and inflexible (Holzinger et al. 2010). It is particularly interesting that in *K.* *crenulatum* a high negative osmotic potential of—2.09 MPa has been determined by incipient plasmolysis (equivalent to an osmolarity of 961 mOsm kg^−1^), which substantially contributes to its water-holding capacities (Kaplan et al. 2012). The ultrastructural appearance upon treatment with sorbitol leads to a condensed cytoplasm similar to that in the desiccation experiments. The cell walls, however, do not shrink in the hyperosmotic solutions, but remain connected with the plasmolysed cytoplasm via Hechtian strands (Kaplan et al. 2012). The osmotic potential of semi-terrestrial *Zygnema* is less negative in younger developmental stages, but increases upon the formation of akinetes (Kaplan et al. 2013). In nature, these akinete stages are found only in late summer (e.g., Holzinger et al. 2009), thus providing the capacity to survive desiccation.

## Protective strategies against desiccation in alpine biological soil crust algae

Eukaryotic algae in BSCs have evolved avoidance and protection strategies to maintain integrity under unfavorable water-potential conditions. So far, little is understood on the community level, but self-protection may be important, as the vertically lower-positioned organisms of a soil crust may not even be exposed to water stress due to the water-holding capacities of the organisms on top and in the crust matrix. A biochemical protection strategy is the production of osmotically active carbohydrates such as polyols, generated particularly by green algae from the Trebouxiophyceae (e.g., Gustavs et al. 2010). However, these compounds are lacking in reasonable concentrations in Klebsormidiophyceae, which are the major component organisms in alpine BSCs (Kaplan et al. 2012).

An organized ‘shutdown’ of PSII occurs during desiccation in BSC algae (Karsten et al. 2010; Karsten and Holzinger 2012). Dynamic photoinhibition has recently been confirmed for several species of desert and aquatic green algae (Lunch et al. 2013). Although photoprotective mechanisms in those green algae that have been investigated are similar, the mechanisms exhibit lineage-specific differences. De-epoxidation of xanthophyll-cycle pigments paralleled light-induced changes in nonphotochemical quenching for species of Klebsormidiophyceae and Trebouxiophyceae, but not Zygnematophyceae, indicating that the pigments involved can contribute to photoprotection, although to different degrees in different lineages (Lunch et al. 2013). When considering that dehydration usually occurs during long periods of sunshine, an overlap with protective and repair strategies against UVR, as described above, occurs.

## Conclusions

Green algae are abundant in alpine BSCs of the Alps. Due to the spatial structure of the soil crusts, protection against direct sunlight including UV-B can be expected, which together with sufficient moisture will assure the long-term survival of these organisms, often under harsh environmental conditions. Since the meteorological data clearly indicate the existence of highly variable seasonal and diurnal fluctuations in radiation, sunshine duration, precipitation and air temperature (Körner 2003), it can be assumed that dehydration will affect the alpine soil crust organisms on a short-term rather than on a long-term scale. Alpine BSC green algae are excellent model systems to study and understand the protective mechanisms against UVR and desiccation. Certain algae contain the capacity to adapt in the long run to their environment, which implies that they could also function as good indicator organisms. This is important in terms of any changes in precipitation or temperature that might be associated with the future scenarios of climate change. It would be particularly interesting to study if, e.g., desiccation-tolerant green algae replace non-desiccation-tolerant ones in certain habitats.

With new developments in genomics, proteomics and metabolomics, the underlying biosynthetic and regulatory pathways can be elucidated. Such studies are urgently needed to provide a deeper insight into the mechanisms involved in the astonishing stress tolerance of these organisms.

## References

[CR1] Aigner S, Remias D, Karsten U, Holzinger A (2013). Unusual phenolic compounds contribute to the ecophysiological performance in the purple-colored green alga *Zygogonium ericetorum* (Zygnematophyceae, Streptophyta) from a high-alpine habitat. J Phycol.

[CR2] Allakhverdiev SI, Kreslavski VD, Klimov VV, Los DA, Carpentier R, Mohanty P (2008). Heat stress: an overview of molecular responses in photosynthesis. Photosynth Res.

[CR3] Asada K, Foyer CH, Mullineaux PM (1994). Production and action of active oxygen species in photosynthetic tissues. Causes of photooxidative stress and amelioration of defence systems in plants.

[CR4] Bandaranayake WM (1998). Mycosporines: are they nature’s sunscreens?. Nat Prod Rep.

[CR5] Belnap J, Lange OL (2001). Biological soil crusts: structure, function and management.

[CR6] Bertsch A (1966). CO_2_ Gaswechsel der Grünalge *Apatococcus lobatus*. Planta.

[CR7] Bischof K, Hanelt D, Wiencke C (2000). Effects of ultraviolet radiation on photosynthesis and related enzyme reactions of marine macroalgae. Planta.

[CR8] Bischof K, Gómez I, Molis M, Hanelt D, Karsten U, Lüder U, Roleda MY, Zacher K, Wiencke C (2006). Ultraviolet radiation shapes seaweed communities. Rev Environ Sci Biotechnol.

[CR9] Blumthaler M, Lütz C (2012). Solar radiation of the high alps. Plants in Alpine regions.

[CR10] Blumthaler M, Ambach W (1990). Indication of increased solar ultraviolet-B radiation flux in Alpine regions. Science.

[CR11] Blumthaler M, Ambach W, Möller R (1996). Increase in solar UV radiation with altitude. J Photochem Photobiol.

[CR12] Büdel B, Buscot F, Varma A (2005). Microorganisms of biological crusts on soil surface. Microorganisms in soils: roles in genesis and functions, soil biology.

[CR13] Büdel B, Lüttge U, Beck E, Bartels D (2011). Eucaryotic algae. Plant desiccation tolerance, ecological studies, vol 215.

[CR14] Campbell D, Eriksson MJ, Öquist G, Gustafsson P, Clarke AK (1998). The cyanobacterium *Synechococcus* resists UV-B by exchanging photosystem II reaction-center D1 proteins. Proc Nat Acad Sci USA.

[CR15] Cardon ZG, Gray DW, Lewis LA (2008). The green algal underground: evolutionary secrets of desert cells. Bioscience.

[CR16] De Winder B, Matthijs HCP, Mur LR (1990). The effect of dehydration and ion stress on carbon dioxide fixation in drought-tolerant phototrophic microorganisms. FEMS Microb Ecol.

[CR17] Elbert W, Weber B, Burrows S, Steinkamp J, Büdel B, Andreae MO, Pöschl U (2012). Contribution of crytogamic covers to the global cycles of carbon and nitrogen. Nat Geosci.

[CR18] Elstner EF (1990). Der Sauerstoff.

[CR19] Ettl H, Gärtner G (1995). Syllabus der Boden-, Luft- und Flechtenalgen.

[CR20] Evans RD, Johansen JR (1999). Microbiotic crusts and ecosystem processes. Crit Rev Plant Sci.

[CR21] Franklin LA, Forster FM (1997). The changing irradiance environment: consequences for marine macrophyte physiology, productivity and ecology. Eur J Phycol.

[CR22] Garcia-Pichel F, Loza V, Marusenko Y, Mateo P, Potrafka RM (2013). Temperature drives the continental-scale distribution of key microbes in topsoil communities. Science.

[CR23] Gärtner G (2004). ASIB—the culture collection of algae at the Botanical Institute, Innsbruck, Austria. Nova Hedwig.

[CR24] Gasulla F, deNova PG, Esteban-Carrasco A, Zapata JM, Barreno E, Guéra A (2009). Dehydration rate and time of desiccation affect recovery of the lichenic algae *Trebouxia erici*: alternative and classical protective mechanisms. Planta.

[CR25] Gray DW, Lewis LA, Cardon ZG (2007). Photosynthetic recovery following desiccation of desert green algae (Chlorophyta) and their aquatic relatives. Plant Cell Environ.

[CR26] Gustavs L, Eggert A, Michalik D, Karsten U (2010). Physiological and biochemical responses of aeroterrestrial green algae (Trebouxiophyceae) to osmotic and matric stress. Protoplasma.

[CR27] Gustavs L, Görs M, Karsten U (2011). Polyols as chemotaxonomic markers to differentiate between aeroterrestrial green algae (Trebouxiophyceae, Chlorophyta). J Phycol.

[CR28] Harm W (1980). Biological effects of ultraviolet radiation.

[CR30] Holzinger A, Karsten U (2013). Desiccation stress and tolerance in green algae: consequences for ultrastructure, physiological, and molecular mechanisms. Front Plant Sci.

[CR31] Holzinger A, Lütz C (2006). Algae and UV irradiation: effects on ultrastructure and related metabolic functions. Micron.

[CR32] Holzinger A, Wasteneys G, Lütz C (2007). Investigating cytoskeletal function in chloroplast protrusion formation in the arctic-alpine plant *Oxyria digyna*. Plant Biol.

[CR33] Holzinger A, Roleda MY, Lütz C (2009). The vegetative arctic green alga *Zygnema* is insensitive to experimental UV exposure. Micron.

[CR34] Holzinger A, Tschaikner A, Remias D (2010). Cytoarchitecture of the desiccation-tolerant green alga *Zygogonium ericetorum*. Protoplasma.

[CR35] Holzinger A, Lütz C, Karsten U (2011). Desiccation stress causes structural and ultra-structural alterations in the aeroterrestrial green alga *Klebsormidium crenulatum* (Klebsormidiophyceae, Streptophyta) isolated from an alpine soil crust. J Phycol.

[CR36] Hoppert M, Reimer R, Kemmling A, Schröder A, Günzl B, Heinken T (2004). Structure and reactivity of a biological soil crust from a xeric sandy soil in Central Europe. Geomicrobiol J.

[CR37] Kaplan F, Lewis LA, Wastian J, Holzinger A (2012). Plasmolysis effects and osmotic potential of two phylogenetically distinct alpine strains of *Klebsormidium* (Streptophyta). Protoplasma.

[CR38] Kaplan F, Lewis LA, Herburger K, Holzinger A (2013). Osmotic stress in the arctic and antarctic green alga *Zygnema* sp. (Zygnematales, Streptophyta): effects on photosynthesis and ultrastructure. Micron.

[CR39] Karsten U, Holzinger A (2012). Light, temperature, and desiccation effects on photosynthetic activity, and drought-induced ultrastructural changes in the green alga *Klebsormidium dissectum* (Streptophyta) from a high alpine soil crust. Microb Ecol.

[CR40] Karsten U, Lembcke S, Schumann R (2007). The effects of ultraviolet radiation on photosynthetic performance, growth and sunscreen compounds in aeroterrestrial biofilm algae isolated from building facades. Planta.

[CR41] Karsten U, Lütz C, Holzinger A (2010). Ecophysiological performance of the aeroterrestrial green alga *Klebsormidium crenulatum* (Charophyceae, Streptophyta) isolated from an alpine soil crust with an emphasis on desiccation stress. J Phycol.

[CR42] Karsten U, Pröschold T, Mikhailyuk T, Holzinger A (2013). Photosynthetic performance of different genotypes of the green alga *Klebsormidium* sp. (Streptophyta) isolated from biological soil crusts of the Alps. Algol Stud.

[CR43] Kirst GO (1990). Salinity tolerance of eukaryotic marine algae. Annu Rev Plant Physiol Plant Mol Biol.

[CR44] Körner C (2003). Alpine plant life—functional plant ecology of high mountain ecosystems.

[CR45] Krause GH, Weiss E (1991). Chlorophyll fluorescence and photosynthesis, the basics. Annu Rev Plant Physiol Plant Mol Biol.

[CR46] Larcher W (2003). Physiological plant ecology: ecophysiology and stress physiology of functional.

[CR47] Larcher W, Lütz C (2012). Bioclimatic temperatures in the high Alps. Plants in Alpine regions.

[CR48] Larcher W, Wagner J (2009). High mountain bioclimate: temperatures near the ground recorded from the timber-line to the nival zone in the Central Alps. Contrib Nat Hist.

[CR49] Lewis LA, Seckbach J (2007). Chlorophyta on land: independent lineages of green eukaryotes from arid lands. Algae and cyanobacteria in extreme environments.

[CR50] Lewis LA, Lewis PO (2005). Unearthing the molecular phylodiversity of desert soil green algae (Chlorophyta). Syst Biol.

[CR51] Lois R, Buchanan BBN (1994). Severe sensitivity to ultraviolet radiation in an *Arabidopsis* mutant deficient in flavonoid accumulation: II. Mechanisms of UV-resistance in *Arabidopsis*. Planta.

[CR52] Lunch CK, LaFountain AM, Thomas S, Frank HA, Lewis LA, Cardon ZG (2013). The Xanthophyll cycle and NPQ in diverse desert and aquatic green algae. Photosynth Res.

[CR53] Lütz C, Engel L (2007). Changes in chloroplast ultrastructure in some high-alpine plants: adaptation to metabolic demands and climate?. Protoplasma.

[CR54] Morison MO, Sheath RG (1985). Responses to desiccation stress by *Klebsormidium rivulare* (Ulotrichales, Chlorophyta) from a Rhode Island stream. Phycologia.

[CR55] Pichrtová M, Remias D, Lewis LA, Holzinger A (2013). Changes in phenolic compounds and cellular ultrastructure of Arctic and Antarctic strains of *Zygnema* (Zygnematales, Streptophyta) after exposure to experimentally enhanced UV to PAR ratio. Microb Ecol.

[CR56] Reisigl H (1964). Zur Systematik und Ökologie alpiner Bodenalgen. Österr Bot Z.

[CR57] Remias D, Lütz C (2012). Cell structure and physiology of alpine snow and ice algae. Plants in Alpine regions.

[CR58] Reynolds R, Belnap J, Reheis M, Lamothe P, Luiszer F (2001). Aeolian dust in Colorado Plateau soils: nutrient inputs and recent change in source. Proc Natl Acad Sci USA.

[CR59] Šabacká M, Elster J (2006). Response of cyanobacteria and algae from Antarctic wetland habitats to freezing and desiccation stress. Polar Biol.

[CR60] Škaloud P, Rindi F (2013). Ecological differentiation of cryptic species within an asexual protist morphospecies: a case study of filamentous green alga *Klebsormidium* (Streptophyta). J Eukaryot Microbiol.

[CR61] Tschaikner A, Ingolic E, Gärtner G (2007). Observations in a new isolate of *Coelastrella terrestris* (Reisigl) Hegewald & Haganata (Chlorophyceae, Seenedesmaceae) from alpine soil (Tyrol, Austria). Phyton.

[CR62] Tschaikner A, Gärtner G, Kofler W (2008). *Coelastrella aeroterrestrica* sp. nov. (Chlorophyta, Scenedesmoideae)—a new, obviously often overlooked aeroterrestrial species. Algol Stud.

[CR63] Türk R, Gärtner G, Belnap J, Lange OL (2001). Biological soil crusts in the subalpine, alpine, and nival areas in the Alps. Biological soil crusts: structure, function and management.

[CR64] Vass I, Pessarakli M (1997). Adverse effects of UV-B light on the structure and function of the photosynthetic apparatus. Handbook of photosynthesis.

[CR65] Vinatzer G (1975). Neue Bodenalgen aus den Dolomiten. Plant Syst Evol.

[CR66] Wieners PC, Mudimu O, Bilger W (2012). Desiccation-induced non-radiative dissipation in isolated green lichen algae. Photosynth Res.

